# Two-pore domain potassium channels in the adrenal cortex

**DOI:** 10.1007/s00424-014-1628-6

**Published:** 2014-10-23

**Authors:** Sascha Bandulik, Philipp Tauber, Enzo Lalli, Jacques Barhanin, Richard Warth

**Affiliations:** 1 Medical Cell Biology, University of Regensburg, Universitaetsstrasse 31, 93053 Regensburg, Germany; 2 Institut de Pharmacologie Moléculaire et Cellulaire, CNRS, Université de Nice Sophia Antipolis, 660 Route des Lucioles, 06560 Valbonne, France; 3 LP2M-CNRS-UNS UMR 7370, Faculté de Médecine, Université de Nice Sophia Antipolis, 28 Avenue de Valombrose, 06107 Nice Cedex 2, France; 4 Laboratories of Excellence, Ion Channel Science and Therapeutics, Nizza, France

**Keywords:** KCNK2, KCNK3, KCNK9, TASK, TREK, Aldosterone

## Abstract

The physiological control of steroid hormone secretion from the adrenal cortex depends on the function of potassium channels. The “two-pore domain K^+^ channels” (K2P) TWIK-related acid sensitive K^+^ channel 1 (TASK1), TASK3, and TWIK-related K^+^ channel 1 (TREK1) are strongly expressed in adrenocortical cells. They confer a background K^+^ conductance to these cells which is important for the K^+^ sensitivity as well as for angiotensin II and adrenocorticotropic hormone-dependent stimulation of aldosterone and cortisol synthesis. Mice with single deletions of the *Task1* or *Task3* gene as well as *Task1*/*Task3* double knockout mice display partially autonomous aldosterone synthesis. It appears that TASK1 and TASK3 serve different functions: TASK1 affects cell differentiation and prevents expression of aldosterone synthase in the zona fasciculata, while TASK3 controls aldosterone secretion in glomerulosa cells. TREK1 is involved in the regulation of cortisol secretion in fasciculata cells. These data suggest that a disturbed function of K2P channels could contribute to adrenocortical pathologies in humans.

## Introduction

The distinct zones of the adrenal cortex produce different steroid hormones, which regulate several important physiological functions. The mineralocorticoid aldosterone is synthesized by the outermost cell layer (zona glomerulosa) beneath the capsule of the adrenal gland. Glucocorticoids are produced in the zona fasciculata that consists of column-like organized cells below zona glomerulosa. In humans, but not in rodents, the innermost zona reticularis cells produce androgenic steroid hormones. In cells of all three zones of the adrenal cortex, the function of K^+^ channels is an important determinant for controlling hormone secretion, cell differentiation, proliferation, and possibly apoptosis. This review aims to discuss the physiology and pathophysiology of “two-pore domain K^+^ channels” (K2P) in the adrenal cortex, especially in aldosterone-producing glomerulosa cells.

Aldosterone controls the extracellular fluid and salt balance by stimulation of sodium reabsorption and potassium secretion in the distal nephron of the kidney, in the distal colon, and in sweat glands. By controlling water and salt balance and by direct effects on the cardiovascular system, aldosterone has a major impact on blood pressure control. So-called primary aldosteronism is characterized by inappropriately high plasma aldosterone levels due to autonomous aldosterone synthesis. Inadequately high aldosterone secretion is believed to be causal for about 3 % of the cases of arterial hypertension [97]. Additionally, aldosterone contributes to cardiac fibrosis, cardiovascular dysfunction, and progressive kidney disease [59, 111]. The relevance of aldosterone as clinical risk factor has been stressed by clinical trials (Aldosterone Evaluation Study (RALES); EPlerenone HEart failure and SUrvival Study (EPHESUS)) [16, 148]. Therefore, understanding the physiology and pathophysiology of aldosterone synthesis is of great relevance for the diagnosis and treatment of arterial hypertension and cardiovascular disease.

It is known for a long time that the regulation of aldosterone synthesis strongly depends on the modulation of the membrane potential of glomerulosa cells. Also cortisol synthesis appears to be stimulated by depolarization of the plasma membrane. The membrane potential of resting adrenocortical cells is mainly determined by the function of K^+^ channels. Accordingly, the disturbed function of adrenal K^+^ channels has pathological consequences for the regulation of steroid hormone production, and it may lead to excessive proliferation of adrenocortical cells.

## Adrenocortical K^+^ channels

The resting membrane potential of human glomerulosa cells is set by a number of K^+^ channels, particularly of the K2P family, which are highly expressed among species (Table 1). In rodents, two members of the TWIK-related acid sensitive K^+^ (Task) family (Task1 and Task3) were shown to play an important role in the regulation of aldosterone secretion and adrenocortical cell differentiation [7, 28, 34, 51, 55, 103]. TWIK-related K^+^ channel 1 (Trek1) is important for the normal function of the bovine adrenal cortex [38, 41]. The role of K2P channels in the human adrenal gland is still under investigation. Several studies indicate that K2P channels contribute to the physiological control of aldosterone synthesis in human adrenocortical cells. TASK1 is strongly expressed in the human adrenal cortex [22] and in the human adrenocortical NCI-H295R cell line [96]. Silencing of TASK1 expression stimulates aldosterone secretion in NCI-H295R cells [96]. TREK1 and TASK3 K^+^ channels are also expressed in NCI-H295R cells, albeit on a much lower level than TASK1 [96]. Inactivation of TREK1 and TASK3 depolarizes the membrane potential of NCI-H295R cells. However, the amount of this depolarization is likely small, because aldosterone production is not significantly increased [14]. Decreased expression of TASK2 was found in adrenal adenomas [5, 72], and suppression of TASK2 activity in NCI-H295R cells increased aldosterone synthesis [72]. TREK1 was shown to dominate the K^+^ conductance of human fasciculata cells [39]. Most of the knowledge about the functional role of Task K^+^ channels has been obtained by phenotyping different knockout mouse models. The following paragraphs aim at providing a comprehensive overview of the specific role and relevance of K2P channels for the regulation of steroid hormone synthesis and zonal differentiation of the adrenal gland.

**Table 1:** Adrenal K^+^ channels

Channel	Expression	Function	Pathology	Reference
TASK1 (KCNK3)	Mouse: ZG>ZF>inner adrenal cortex	Maintenance and regulation of membrane potential of adrenocortical and medullary cells; Inhibition by Ang-II and endothelin-1; prevention of Cyp11b2 expression in ZF of ♀ mice	*Task1* ^−/−^ mouse: sex-dependent hyperaldosteronism due to ectopic Cyp11b2 expression in ZF	[22, 27, 28, 55, 62, 63, 86, 96, 121]
	Rat: ZG, medulla		Human: pulmonary hypertension	
	Guinea pig: medulla			
	Human: ZG>ZF>ZR (unpublished data), aldosterone-producing adenoma, adrenocortical cell line (NCI-H295R cells)			
TASK3 (KCNK9)	Mouse: ♂ ZG, ZF; ♀ ZG	Maintenance and regulation of membrane potential of adrenal cortex; inhibition by Ang-II; probably heterodimers with Task1	Mild hyperaldosteronism in adult *Task3* ^−/−^ mice; severe hyperaldosteronism in neonatal *Task3* ^−/−^ mice	[6, 9, 14, 22, 25, 26, 28, 51, 83, 103]
	Rat: ZG			
	Human: low adrenal expression compared to TASK1 and TASK2; adrenocortical cell line (NCI-H295R cells)		Human: hypotension and mental retardation	
TASK2 (KCNK5)	Mouse: inner adrenal cortex (unpublished data)	Probably maintenance and regulation of membrane potential of adrenal cortex; expression of a dominant negative TASK2 mutant in NCI-H295R cells stimulated aldosterone synthesis	Decreased expression in aldosterone-producing adenomas	[5, 22, 26, 72]
	Human: adrenal cortex			
TREK1 (KCNK2)	Bovine: ZG, ZF	Maintenance and regulation of membrane potential of adrenal cortex; inhibition by Ang-II, ACTH and vasopressin; expression induced by ACTH and cAMP		[22, 36–39, 41, 80–82]
	Human: ZF			
	Mouse: unknown			
KCNJ5 (Kir3.4/GIRK4)	Human: ZG>ZF, aldosterone-producing adenoma, adrenocortical cell line (NCI-H295R cells)	Function in ZG still unknown; G_ßγ_ activated; Ang-II reduced KCNJ5 expression	Somatic mutations in 30–40 % of aldosterone-producing adenomas; germline mutations in patients with familial hyperaldosteronism type III	[5, 22, 69, 98]
	Pig: ZG (unpublished data)			
KCNQ1/KCNE1	Mouse: adrenal cortex	Repolarization of membrane potential; KCNE1 as regulatory subunit; voltage activated	*Kcnq1* ^−/−^ mouse: hypoaldosteronism	[4, 22, 116, 138]
	Human: adrenal cortex (K^+^ channel with the highest level of expression), adrenocortical cell line (NCI-H295R cells)		Kcne1^−/−^ mouse: hyperaldosteronism under hyperkalemia	
Maxi K (KCNMA1/KCNMB1)	Mouse: ZG and medulla	Repolarization of membrane potential; Ca^2+^ and voltage activated; channel activation by ANP inhibits aldosterone production; KCNMB1 as regulatory subunit	*Kcnma1* ^−/−^ mouse: hyperaldosteronism	[22, 46, 48, 118, 143, 146, 147]
	Human: adrenal cortex		*Kcnmb1* ^−/−^ mouse: hyperaldosteronism	

*ZG* zona glomerulosa, *ZF* zona fasciculata

## Stimulation of aldosterone secretion

**Fig. 1:**
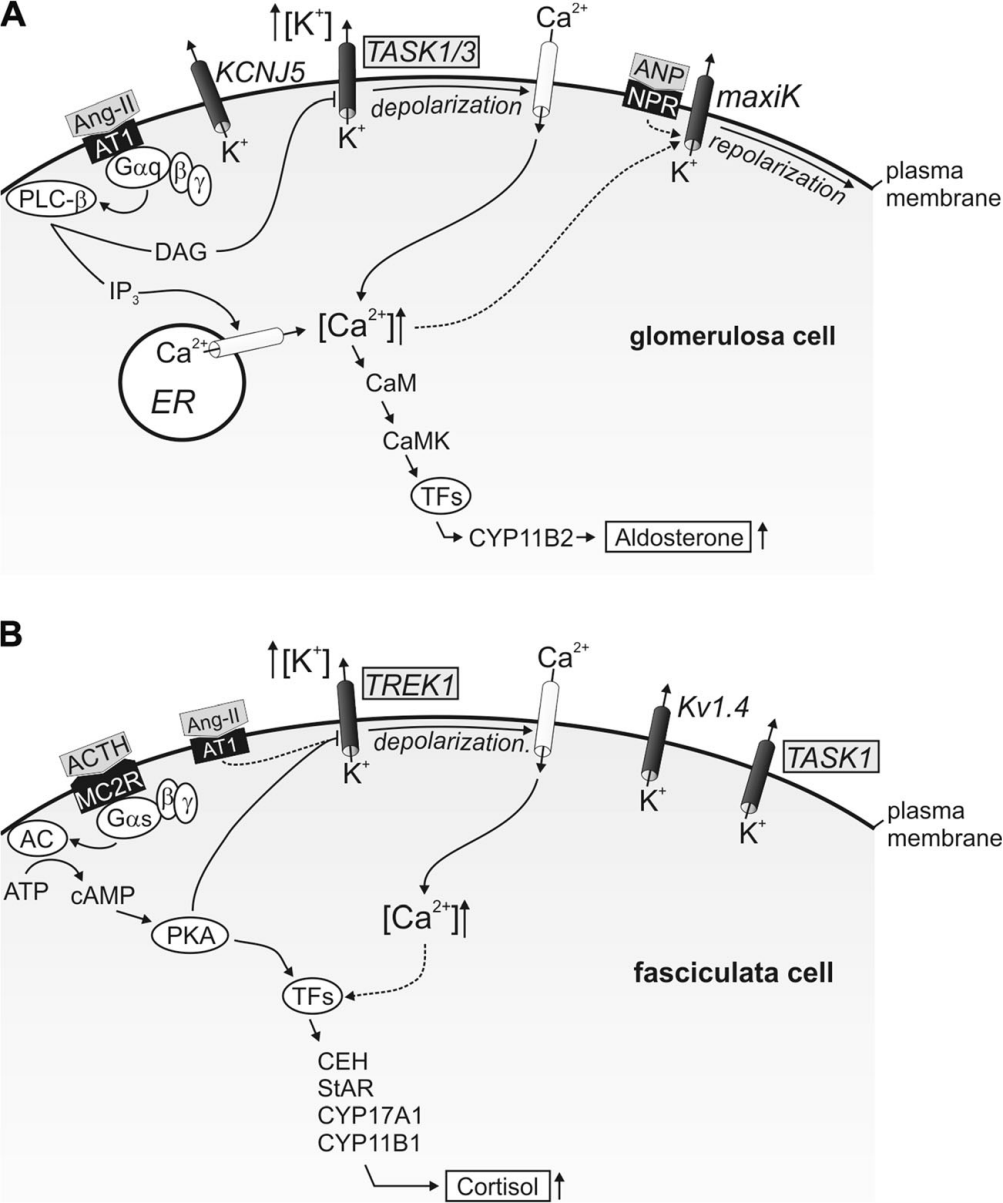
Simplified models for the regulation of aldosterone synthesis in zona glomerulosa cells (**a**) and of cortisol synthesis in zona fasciculata cells (**b**). **a** Stimulatory action of Ang-II and increased plasma K^+^ concentration on aldosterone synthesis depends on membrane voltage depolarization and on increased cytosolic Ca^2+^. G-Protein-dependent activation of phospholipase-C (PLC-ß) via binding of Ang-II to angiotensin receptor 1 (AT1) leads to generation of inositol-triphosphate (IP_3_) and diacylglycerol (DAG). IP_3_ stimulates Ca^2+^ store release from the endoplasmatic reticulum (ER). DAG-dependent inhibition of TASK1 and TASK3 K^+^ channels or a high K^+^-induced shift of the Nernst potential depolarize the membrane. The depolarization activates voltage-dependent Ca^2+^ channels. Ca^2+^-calmodulin activates CaM-Kinases, and this leads to activation of transcription factors (TFs) and increased transcription of CYP11B2 (aldosterone synthase). MaxiK K^+^ channels are activated by the atrial natriuretic peptide (ANP), which binds to the natriuretic peptide receptor (NPR), or by increases of cytosolic Ca^2+^. MaxiK channels repolarize glomerulosa cells and decrease aldosterone synthesis. KCNJ5 K^+^ channels are highly expressed in human glomerulosa cells, but seem to be inactive under control conditions. **b** The stimulatory effect of ACTH on cortisol synthesis depends on cAMP-dependent signaling, but also involves membrane depolarization and increased cytosolic Ca^2+^. ACTH binds to the melanocortic-2-receptor (MC2R) and leads to activation of a G_αs_-protein that stimulates adenylate cyclase (AC). cAMP-activated protein kinase A (PKA) activates transcription factors (TFs) inducing transcription of steroidogenic enzymes. These enzymes are required for cortisol synthesis (e.g., CHE: cholesterolester hydrolase, StAR: steroidogenic acute regulated protein, CYP17A1, CYP11B1). PKA also inhibits TREK1 K^+^ channels, depolarizes the membrane and promotes Ca^2+^ influx and consecutive activation of transcription factors. TREK1 is also inhibited by Ang-II. Additionally, TASK1 and Kv1.4 K^+^ channels are expressed in fasciculata cells

Aldosterone synthesis in adrenal zona glomerulosa cells is mainly stimulated by angiotensin II (Ang-II), by high plasma K^+^ concentrations, and, to a minor extent, by the adrenocorticotropic hormone (ACTH). For the stimulation of aldosterone synthesis by Ang-II or hyperkalemia, modulation of the membrane potential is an early and critical early event in the cellular signaling cascade (Fig. 1). Therefore, precise control of the membrane voltage is very important. A large proportion of the K^+^ channels that determine the resting membrane voltage of glomerulosa cells are constitutively open, e.g., “background” or “leak” K^+^ channels of the K2P family. Due to the high K^+^ conductance, the resting membrane potential of glomerulosa cells is hyperpolarized (−80 mV), close to the K^+^ equilibrium potential. An increase of the extracellular K^+^ concentration, according to Nernst’s equation, leads to a positive shift of the K^+^ equilibrium potential and to a depolarization. By this mechanism, glomerulosa cells are able to sense changes of plasma K^+^ concentration, reminiscent of K^+^-selective electrodes. Upon depolarization of the membrane, voltage-dependent T-type and L-type Ca^2+^ channels are activated, thereby translating the membrane depolarization into a rise of the intracellular Ca^2+^ activity. High intracellular Ca^2+^ activity, via binding to calmodulin and activation of calmodulin-dependent kinases, induces transcription of particular enzymes needed for aldosterone synthesis, e.g., aldosterone synthase (CYP11B2), and steroidogenic acute regulatory protein (StAR) [23]. Aldosterone synthase catalyzes the final three-step reaction from 11-deoxycorticosterone to aldosterone, and it is considered to be the rate-limiting enzyme of aldosterone synthesis. StAR is a transport protein facilitating the shuttling of cholesterol from the outer to the inner mitochondrial membrane where cholesterol is converted to pregnenolone, a precursor of steroid hormones.

The mechanism by which Ang-II depolarizes the membrane is different from the one of high extracellular K^+^. Ang-II depolarizes the plasma membrane by inhibiting background K2P K^+^ channels. The molecular mechanism of the Ang-II-mediated K^+^ channel inhibition was a matter of debate for a long time [19, 79, 87, 121] but was solved only recently. Binding of Ang-II to the AT_1_ receptor activates phospholipase-C via G_αq_-proteins. By cleavage of phosphatidylinositol 4,5-bisphosphate (PIP_2_), phospholipase-C generates diacylglycerol (DAG) and inositol-triphosphate (IP_3_). Interestingly, it appears that DAG acts as a K2P channel-inhibiting factor leading to a strong decrease of the fractional K^+^ conductance and depolarization of the membrane [145]. Similar to the high K^+^-induced depolarization, the Ang-II-induced depolarization activates voltage-gated Ca^2+^ channels and leads to Ca^2+^ influx [1, 58, 137]. In addition, Ang-II facilitates the opening of Ca^2+^ channels by lowering the voltage threshold for activation [84], and it induces a release of Ca^2+^ from IP_3_-sensitive intracellular Ca^2+^ stores [129]. The cytosolic rise of Ca^2+^ is further amplified by store-operated Ca^2+^ entry [95, 106, 112, 139].

Besides its effects on gene transcription of steroidogenic enzymes, intracellular Ca^2+^ induces a very fast non-transcriptional stimulation of aldosterone secretion. In mice, a strong peak of aldosterone secretion is observed within 10 min after injection of Ang-II [130], although aldosterone is not believed to be stored within the cells. Probably, a variety of non-genomic effects of Ca^2+^ underlies this fast response, e.g., an increase of intracellular Ca^2+^ stimulates the activity of StAR [21, 76] and is paralleled by an influx of Ca^2+^ into the mitochondria [144]. A rise in mitochondrial Ca^2+^ enhances the availability of NAPDH, a cofactor of several steroidogenic enzymes [107, 113]. As a negative feedback, an increase of intracellular Ca^2+^ activates Ca^2+^-regulated K^+^ channels, which hyperpolarize the membrane, thereby preventing overwhelming aldosterone secretion. Chronic stimulation of aldosterone production elicits trophic effects on the adrenal gland. Long-term treatment of rats with Ang-II or a low Na^+^ diet induces proliferation and hypertrophy of aldosterone-producing glomerulosa cells [88, 114].

ACTH acts at least in two ways on adrenocortical cells; it acutely stimulates cortisol (and aldosterone) secretion, and it promotes cell proliferation and differentiation of glomerulosa and fasciculata cells [131, 134]. With regard to aldosterone secretion, the acute effect of ACTH is mainly mediated via an increased supply of cholesterol, the precursor of steroid hormones [54]. In addition, ACTH facilitates voltage-gated Ca^2+^ channel opening probably by direct phosphorylation and indirectly via inhibition of K^+^ channels leading to depolarization [38, 39, 41, 44, 81, 124, 136]. In bovine and human fasciculata cells, ACTH is a negative regulator of TREK1. TREK1 is also expressed in bovine cells from zona glomerulosa and in human adrenocortical NCI-H295R cells [14, 36, 80]. Inactivation of TREK1 in NCI-H295R cells induces a depolarization [14]. By contrast, native primary cultured human glomerulosa cells do not show K^+^ currents resembling TREK1 [102]. Trek1 is also expressed in mouse adrenocortical cells (UniGene data), but an adrenal phenotype of the *Trek1* knockout mouse has not yet been reported [56, 91, 142].

## Adrenocortical cells are excitable

Cultured primary cells or adrenocortical cell lines display a very high K2P K^+^ channel activity which is inhibited by Ang-II. The inhibition of these K^+^ channels depolarizes the membrane and triggers the activation of voltage-gated Ca^2+^ channels and hormone synthesis. Dispersed and cultured adrenocortical cells are considered non-excitable cells; they usually do not display action potentials. However, experiments under more physiological conditions, e.g., on fresh adrenal slices, have disclosed that native adrenocortical cells are excitable with rapid oscillations of membrane voltage and intracellular Ca^2+^ [58, 103, 109, 115]. In slice preparations, Ang-II-induced Ca^2+^ waves propagate from cell to cell suggesting functional coupling of adrenocortical cells via gap junctions [103]. Most likely, oscillatory activity of K^+^ and Ca^2+^ channels and Ca^2+^-transporting systems is the molecular correlate of these oscillations. The absence of Ca^2+^ oscillations in most adrenocortical cell lines and adrenal primary cells suggests that adrenal-specific cell differentiation and electrical properties are not sufficiently preserved in these “model systems”.

What is the possible role of K^+^ channels without oscillatory activity, such as K2P, in excitable adrenocortical cells? As mentioned above, the activity of K^+^ channels is essential for hyperpolarizing the membrane voltage at resting conditions, and it allows glomerulosa cells to act as sensors of extracellular K^+^. Constitutively, active K^+^ channels ensure the hyperpolarized membrane voltage that is required for hyperpolarization-dependent extrusion of Ca^2+^, e.g., via Na^+^/Ca^2+^ exchangers. Moreover, these K^+^ channels determine the level of excitability of adrenocortical cells and the frequency of action potentials, reminiscent of the role of K_ATP_ K^+^ channels in insulin-secreting cells. In pancreatic beta cells, K_ATP_ channel inhibition is induced by rises of the ATP/ADP ratio and results in membrane depolarization, action potentials, and oscillations of intracellular Ca^2+^ [60]. In a similar way, Ca^2+^ oscillations in adrenal glomerulosa cells are triggered by Ang-II-induced inhibition of K2P channels. This function of adrenal K2P channels is important for the regulation of aldosterone synthesis under physiological and pathophysiological conditions [58].

## Other factors controlling aldosterone secretion

Besides the main regulators (Ang-II, high plasmatic K^+^ concentrations, ACTH), a variety of other mediators modulate aldosterone secretion, e.g., serotonin, endothelin-1 [129]. Over the last decades, the pathways stimulating aldosterone secretion have been in the focus of research activities. Conversely, mechanisms that inhibit the synthesis of aldosterone and prevent excessive secretion are also important e.g., atrial natriuretic peptide (ANP)-induced inhibition of StAR expression [20], ANP-mediated activation of Ca^2+^-dependent MaxiK (KCNMA1) K^+^ channels [46], activation of voltage-gated K^+^ channels such as KCNQ1, as well as Na^+^- and G-protein-dependent activation of Kir3.4 (KCNJ5) K^+^ channels. These K^+^ channels have in common that their activation hyperpolarizes the membrane thereby reducing Ca^2+^ influx and aldosterone secretion. Other regulatory pathways involve the control of Na^+^/K^+^ ATPase activity and of transport systems lowering intracellular Ca^2+^, e.g., Na^+^/Ca^2+^ exchangers (NCX and NCKX) and Ca^2+^ ATPases [11, 133]. In addition to acute effects, a complex network of regulating factors controls cell proliferation, centripetal migration, and differentiation as well as apoptosis of adrenocortical cells [129].

## Regulation of cortisol/corticosterone synthesis

Glucocorticoids affect a plethora of physiological functions, e.g., lipid and glucose metabolism and the immune system. Inappropriately, high cortisol production is a characteristic for Cushing’s syndrome (hypercortisolism) [126]. Cortisol synthesis in fasciculata cells is mainly controlled by ACTH. Binding of ACTH to the G_s_-protein-coupled melanocortin 2 receptor (MC2R) activates adenylate cyclase leading to increased cAMP synthesis. Activated by cAMP, protein kinase A (PKA) phosphorylates several target proteins. Two of these target proteins improve the supply with cholesterol: cholesterol ester hydrolase (CEH) releases cholesterol from esterified cholesterol in intracellular lipid droplets [125], and StAR shuttles cholesterol into the inner mitochondrial membrane. Moreover, PKA phosphorylates transcription factors that stimulate expression of the 11ß-hydroxylase (CYP11B1), the enzyme catalyzing the final step of glucocorticoid synthesis [47, 127]. The central role of PKA signaling for cortisol synthesis was recently underlined by the observation that a somatic gain-of-function mutation of PKA is present in about 50 % of patients with cortisol-producing adrenal adenomas [12, 15, 47, 117]. Another target of PKA is the K2P channel TREK1 that is inhibited by phosphorylation [39]. Due to inhibition of TREK1, ACTH stimulation leads to depolarization of fasciculata cells and activation of voltage-dependent Ca^2+^ channels [39]. One might speculate that chronic TREK1 inhibition contributes to the pathophysiology of cortisol-producing adenoma cells carrying the gain-of-function mutation of PKA. Interestingly, the *Trek1* knockout mouse does not present with obvious signs of Cushing’s syndrome suggesting that Trek1 inhibition increases glucocorticoid secretion only in the presence of other stimulating factors.

Why are such mutations enhancing cAMP-dependent pathways not detected in aldosterone-producing adenomas? Adrenocortical cells undergo a centripetal migration and differentiation starting from capsular and subcapsular stem cells and ending up by apoptosis at the border between the adrenal cortex and medulla. The subcapsular-medullar migration is accompanied by a shift of differentiation from the glomerulosa to the fasciculata cell type [43]. ACTH influences these important processes: ACTH stimulates proliferation and accelerates centripetal differentiation of adrenocortical cells [131]. On the other hand, suppression of ACTH by dexamethasone decreases proliferation and differentiation [131]. Thus, alterations of the cAMP/PKA pathway will most likely effect proliferation, migration, and differentiation of adrenocortical cells. One might speculate that in glomerulosa cells, mutational activation of PKA will probably cause an accelerated differentiation. Thus, adenomas originating from glomerulosa cells with constitutively active PKA might be phenotypical classified as cortisol secreting tumors. Further studies are needed to test this hypothesis.

Besides activating aldosterone secretion, Ang-II also stimulates synthesis of cortisol in bovine and human fasciculata cells [39, 40]. Fasciculata cells display similar oscillations of the membrane potential as it was described for glomerulosa cells [8, 58, 94, 108]. Via inhibition of K2P channels, Ang-II depolarizes fasciculata cells resulting in activation of voltage-dependent Ca^2+^ channels. These synergistic effects of ACTH and Ang-II on cortisol secretion might be of particular importance under stress conditions when a strong increase of cortisol secretion is needed.

## Mutations of ion-transporting membrane proteins are associated with aldosterone-producing adenomas

The regulation of membrane voltage and cytosolic Ca^2+^ activity is central for the physiological control of aldosterone secretion. Disturbed function of proteins controlling membrane voltage and Ca^2+^ homeostasis can cause adrenal diseases. The importance of the control of membrane voltage and intracellular ion composition is exemplified by somatic mutations of K^+^ channels, Ca^2+^ channels, the Na^+^/K^+^-ATPase, and a plasma membrane Ca^2+^-ATPase found in aldosterone-producing adenomas [3]. The most frequently mutated gene is the inwardly rectifying K^+^ channel *Kir3.4* (*KCNJ5*). About 40 % of adrenal adenomas show somatic mutations of *Kir3.4* [13, 42, 122]. These mutations confer a pathological Na^+^ conductance (“gain-of-function”) to the channel that depolarizes the cells and activates autonomous aldosterone synthesis and proliferation [17, 22, 70, 92, 93, 99, 123, 133]. Although these results clearly established the link between *Kir3.4* mutations and hyperaldosteronism, the physiological role of non-mutated Kir3.4 in the adrenal cortex is still largely elusive. The wild-type Kir3.4 channel seems to be inactive in human adrenal cells under resting conditions [66, 70, 133]. Probably, Kir3.4 modulates the membrane potential of human glomerulosa cells after stimulation of aldosterone synthesis and prevents excess secretion of aldosterone [98]. Unfortunately, Kir3.4 function cannot be studied in mice because the channel is not expressed in the mouse adrenal gland (unpublished data).

## Adrenal phenotype of *Task* channel knockout mice

Different knockout mouse models were used to investigate the relevance of Task1 and Task3 K^+^ channels for the adrenal gland [7, 28, 34, 51, 55, 103]. Mice with single deletions of the *Task1* [55] or *Task3* gene [7, 51, 103] as well as *Task1*/*Task3* double knockout mice displayed disturbances of the steroid hormone homeostasis [28]. A common feature of all these models was the partially autonomous aldosterone synthesis. The severity of the phenotype of the mice was dependent on the inactivated gene. Detailed analysis of the different phenotypes revealed that both K^+^ channels, Task1 and Task3, are necessary for normal control of aldosterone synthesis. Moreover, each Task K^+^ channel seems to play a specific role for the sex- and age-dependent regulation of adrenocortical cell function.

## Adrenal phenotype of *Task1*^−/−^ mice

In adrenal glands of rats and mice, Task1 is expressed in cells of zona glomerulosa and zona fasciculata [27, 28, 55]. Besides this adrenal localization, Task1 is also expressed in the brain, the heart, and vascular tissue. The neurological phenotype of *Task1* ^−/−^ mice is rather mild [2, 32, 77, 78, 90]. In addition, Task1 is expressed in the carotid bodies where it is involved in the chemosensory control of breathing [135]. The characterization of the cardiac phenotype of *Task1* ^−/−^ mice [29, 31] as well as genetic studies identifying genetic TASK1 variations associated with arrhythmia [73] reveals the regulatory role of TASK1 channels in the cardiac conduction system. In humans, several mutations of *TASK1* are associated with autosomal dominant pulmonary hypertension [85].

In mice, aldosterone secretion is strongly altered by deletion of the *Task1* gene [55]. Interestingly, the adrenal phenotype of adult mice is restricted to females, which present a severe primary hyperaldosteronism with low plasma renin and hypokalemia. Female *Task1* ^−/−^ mice are not able to adapt their remarkably high aldosterone levels to different salt diets, which normally increase (low Na^+^ or high K^+^ diet) or decrease (high Na^+^ diet) plasma aldosterone. Similar to patients with hyperaldosteronism, female *Task1* ^−/−^ mice develop arterial hypertension. Treatment with the mineralocorticoid receptor blocker canrenoate leads to normalization of the blood pressure corroborating the link between hyperaldosteronism and hypertension in these animals.

According to the model for the regulation of aldosterone synthesis, the deletion of *Task1* leads to cell membrane depolarization, increased cytosolic Ca^2+^ activity, and increased transcription of aldosterone synthase (*Cyp11b2*). Indeed, female *Task1* ^−/−^ mice show increased messenger RNA (mRNA) and protein expression of aldosterone synthase. The histomorphological basis for the sex-specific hyperaldosteronism, however, is surprising. Glomerulosa cells of female *Task1* ^−/−^ mice are devoid of aldosterone synthase (as measured by immunofluorescence). Instead, female *Task1* ^−/−^ show a strong expression of aldosterone synthase in zona fasciculata cells. The pathological localization of aldosterone synthase suggests a profoundly disturbed zonation of the adrenal cortex. But surprisingly, the localization of the glomerulosa marker Dab2 is preserved, and corticosterone synthesis is also normal in *Task1* ^−/−^ mice. Apparently, the “dezonation” is restricted to specific cellular properties such as the ectopic expression of the aldosterone synthase and does not reflect a totally disturbed adrenocortical zonal architecture. Moreover, treatment of female *Task1* ^−/−^ mice with the synthetic glucocorticoid dexamethasone strongly suppressed the hyperaldosteronism. This suppression of aldosterone secretion might be caused by direct effects of dexamethasone on fasciculata cells [52, 67] or via suppression of ACTH. In *Task1* ^−/−^ fasciculata cells, ACTH might act as a permissive factor for the abnormal expression of the aldosterone synthase. This is reminiscent of a glucocorticoid-remediable form of familial hyperaldosteronism (FH-I), which is caused by a *CYP11B1*/*CYP11B2* chimeric gene expressed under the control of ACTH [75]. In which way could ACTH modulate the phenotype of female *Task1* ^−/−^ mice? Via inhibition of Trek1, ACTH probably depolarizes the plasma membrane [38, 39, 41, 81]. In mice lacking *Task1*, the ACTH-induced depolarization could be more pronounced and sufficient to elicit ectopic expression of aldosterone synthase. In addition, effects of ACTH on cell proliferation and differentiation could influence the severity of the phenotype in female *Task1* ^−/−^ mice.

## Age-dependent phenotype of *Task1*^−/−^ mice

Before puberty, the mislocalization of aldosterone synthase in the zona fasciculata was observed in *Task1* ^−/−^ mice of both sexes. After puberty, male *Task1* ^−/−^ mice restored the normal glomerulosa-specific localization of aldosterone synthase and normal plasma aldosterone levels, while female *Task1* ^−/−^ mice maintained the ectopic expression of the aldosterone synthase and the hyperaldosteronism phenotype. The compensation of the *Task1* invalidation in male mice after puberty was probably driven by androgen-dependent mechanisms. Castration of young male *Task1* ^−/−^ mice prevented restoration of glomerulosa-specific localization of aldosterone synthase as seen in adult male *Task1* ^−/−^ mice. Accordingly, treatment of female *Task1* ^−/−^ mice with testosterone led to the disappearance of aldosterone synthase from fasciculata cells and to a normal expression in glomerulosa cells [55]. Different factors possibly contribute to the compensation of the *Task1* deletion in male mice or testosterone treated female mice. Adult male mice exhibit a higher expression of *Task3* [55], *Trek1*, and *Kcnq1* (unpublished data) K^+^ channels than female mice. In addition, Task3 protein expression in male mice was found in zona glomerulosa and zona fasciculata, while it seems to be largely restricted to zona glomerulosa in female mice [103].

## Adrenal phenotype of *Task1*^−/−^/*Task3*^−/−^ double knockout mice

The possible role of Task3 as a compensatory factor for *Task1* deletion in male mice was tested by analysis of the adrenal phenotype of mice with a double knockout of the *Task1* and *Task3* genes [28]. However, male *Task1* ^−/−^/*Task3* ^−/−^ mice do not develop ectopic expression of aldosterone synthase as observed in female *Task1* ^−/−^ mice. Although those male double knockout mice display hyperaldosteronism, while male *Task1* ^−/−^ mice do not, the normal glomerulosa-specific localization of the aldosterone synthase is retained. Apparently, Task3 is not the sole androgen-dependent factor that establishes a normal distribution of aldosterone synthase in the adrenal cortex of mice lacking Task1.

In patch-clamp measurements, native glomerulosa cells from male *Task1* ^−/−^/*Task3* ^−/−^ mice have no Task-like currents and are severely depolarized. As a consequence, plasma aldosterone levels in male *Task1* ^−/−^/*Task3* ^−/−^ mice are increased. Normally, high Na^+^ diet suppresses plasma renin levels and, thereby, aldosterone secretion. In *Task1* ^−/−^/*Task3* ^−/−^ mice, high Na^+^ diet does not lead to the physiological suppression of aldosterone synthesis. In these mice, plasma renin levels are already suppressed under normal diet, and a further suppression by high Na^+^ intake is not possible. These results indicate that Task3 K^+^ channels are needed for a normal control of aldosterone synthesis in glomerulosa cells, but they are not essential for the suppression of aldosterone synthase expression in zona fasciculata.

## Effect of acidosis on aldosterone secretion of *Task* knockout mice

Besides Ang-II and high plasma K^+^, acidosis is known to stimulate aldosterone synthesis [53, 110, 119, 120]. Task1 and Task3 K^+^ channels are inhibited by extracellular acidification [26, 27, 33]. Therefore, Guagliardo et al. hypothesized that *Task1* ^−/−^/*Task3* ^−/−^ mice exhibit an altered response of aldosterone secretion upon NH_4_Cl-induced acidosis [50]. Interestingly, stimulation of aldosterone production by mild acidosis, as observed in wild-type mice, was not completely abrogated in *Task1* ^−/−^/*Task3* ^−/−^ mice. Apparently, Task1 and Task3 channels are not essential for the stimulatory effect of acidosis on aldosterone secretion. Most likely, acidosis has a dual effect; it stimulates the renin/Ang-II system, and it has a direct effect on adrenal K^+^ channels.

## Expression of Dkk3 modulates the adrenal phenotype of male *Task1*^−/−^ mice

The androgen-dependent compensatory mechanism in male *Task1* ^−/−^ mice is presumably complex and probably involves several factors on different levels of the signaling cascade. In order to identify those factors, El Wakil et al. performed a gene chip analysis [34] to investigate potential changes of adrenal mRNA expression in *Task1* ^−/−^ mice with ectopic aldosterone expression. The most appealing differentially regulated factor was dickkopf-3 (Dkk3). Dkk3 is a member of the dickkopf family and modulates the Wnt/ß-catenin pathway, which is involved in the control of glomerulosa cell function and differentiation in mouse adrenal glands [35]. Dkk3 is expressed in the zona glomerulosa of humans and mice [132], and its expression is stimulated by cytosolic Ca^2+^ [34]. The function of Dkk3 is to inhibit aldosterone synthesis [18], and therefore, it could be a factor counterbalancing the hyperaldosteronism of *Task1* ^−/−^ mice. A possible role of Dkk3 for the compensation of the *Task1* deletion in male mice was verified by phenotyping *Task1* ^−/−^/*Dkk3* ^−/−^ double knockout mice [34]. Similar to female *Task1* ^−/−^ mice [55], male *Task1* ^−/−^/*Dkk3* ^−/−^ mice showed increased plasma aldosterone levels, which were not further stimulated by a K^+^-rich diet. The expression of *Cyp11b2* mRNA was increased, but the localization of the aldosterone synthase was still restricted to the zona glomerulosa. Obviously, Dkk3 functions as a repressor of *Cyp11b2* expression in glomerulosa cells, but it is not essential for suppression of aldosterone synthase expression in zona fasciculata in male *Task1* ^−/−^ mice.

## The adrenal phenotype of *Task3*^−/−^ mice

The specific role of Task3 K^+^ channels for adrenocortical function was investigated using two different *Task3* ^−/−^ mouse models [51, 103]. Under high Na^+^ diet, adult *Task3* ^−/−^ animals do not show the physiological suppression of aldosterone secretion and develop salt-sensitive arterial hypertension [51, 103]. What is the explanation for the lack of adaptation to high dietary Na^+^ intake? Normally, high Na^+^ intake leads to a decrease of the renin and Ang-II levels and to a suppression of aldosterone. In *Task3* ^−/−^ mice, aldosterone secretion is partially autonomous and does not require stimulation by renin/Ang-II. Under normal diet, the autonomous component of aldosterone secretion is masked by a compensatory suppression of renin/Ang-II and a reduction of Ang-II-driven aldosterone secretion. At high Na^+^ diet, a further suppression of renin/Ang-II is not possible. With regard to the high Na^+^ intake, aldosterone stays inappropriately high, Na^+^ is retained in a pathological way, and arterial hypertension develops. Accordingly, the aldosterone/renin ratio, a clinical indicator for autonomous aldosterone production, is strongly increased under a control diet and a high Na^+^ diet in *Task3* ^−/−^ mice. In contrast to the ectopic expression of aldosterone synthase in female *Task1* ^−/−^ mice, *Task3* ^−/−^ mice of both sexes display normal localization of aldosterone synthase in zona glomerulosa [103]. Obviously, invalidation of the *Task3* gene affects the physiological control of aldosterone production, but functional differentiation and zonation of the adrenal cortex are maintained.

Interestingly, Guagliardo et al. observed a hyperpolarized membrane voltage in glomerulosa cells of fresh adrenal slices of *Task3* ^−/−^ mice, although Task3 is believed to be an important K^+^ channel of these cells [51]. How can this surprising observation be explained? In contrast to Guagliardo et al., we found primary cultured adrenocortical cells of *Task3* ^−/−^ mice depolarized to −50 mV compared to −80 mV in wild-type cells. However, after stimulation with Ang-II, primary cells of *Task3* ^−/−^ mice did not show the expected depolarization; they hyperpolarized transiently, probably due to enhanced activity of Ca^2+^-activated K^+^ channels (unpublished data). Most likely, the increased activity of Ca^2+^-activated K^+^ channels in glomerulosa cells of *Task3* ^−/−^ mice compensates for the loss of Task3 and masks the electrical phenotype under certain experimental conditions.

To gain further insights into the role of Task3 for adrenal signaling, intracellular Ca^2+^ was measured in freshly prepared adrenal slices [103]. Slices of wild-type mice showed a spontaneous Ca^2+^ oscillation only in a small number of cells, most of the cells were silent. After stimulation with Ang-II or high extracellular K^+^, most of the wild-type cells showed high frequency Ca^2+^ oscillations. By contrast, in slices of *Task3* ^−/−^ mice, glomerulosa cells often showed spontaneous Ca^2+^ oscillations under control conditions, but the stimulatory effects of Ang-II and high extracellular K^+^ were attenuated [103].

From these Ca^2+^ measurements on adrenal slices, we expected impaired aldosterone response of Task3^−/−^ mice in vivo at high K^+^ diet and low Na^+^ diet (the latter increases renin and Ang-II). Surprisingly, glomerulosa cell of *Task3* ^-/-^ mice still showed a normal increase of aldosterone production under low Na^+^ and high K^+^ diets [51, 103]. Despite the impaired effects of Ang-II and high K^+^ in the slice preparation, the adrenal responsiveness towards major stimulatory pathways appears to be preserved in *Task3* ^−/−^ mice, allowing aldosterone to increase normally in response to these strong stimuli. Probably, several compensatory mechanisms act in concert to counterbalance the impaired membrane and Ca^2+^ signaling. For instance, Ca^2+^-independent signaling pathways (e.g., via lipoxygenase and activation of p38-MAPK [49, 100]) could contribute to the preserved Ang-II effect on aldosterone production. Moreover, an increase of the plasma K^+^ concentration could activate K^+^-sensitive adrenomedullary cells which stimulate glomerulosa cells via paracrine factors [10] or via nerve fibers projecting into the adrenal cortex [24].

## Severe hyperaldosteronism in newborn *Task3*^−/−^ mice

The adrenal phenotype of *Task3* ^−/−^ mice is age-dependent [7]. Newborn *Task3* ^−/−^ mice have a more severe hyperaldosteronism than adult mice. In addition, plasma concentrations of other steroid hormones such as corticosterone and progesterone are increased and transcription of steroidogenic enzymes, e.g., aldosterone synthase and hydroxy-β-5-steroiddehydrogenase, 3 β-and steroid β-isomerase 6 (Hsd3b6, an enzyme needed for glomerulosa-specific progesterone synthesis), is enhanced.

A gene chip analysis was performed to identify transcriptionally regulated potential factors and pathways underlying the transient hyperaldosteronism of neonatal *Task3* ^−/−^ mice. This analysis revealed a strong but transient upregulation of renin mRNA in adrenal glands of 1-day-old *Task3* ^−/−^ mice; in 12-day-old animals, renin expression was back to control values. The renin expressing cells were localized in zona fasciculata. Local renin expression in the adrenal gland and in other extra-renal tissues (e.g., in the heart and the eye) is known for a long time [101]. The exact function of the local adrenal renin/Ang-II system is not well understood. It was suggested that local renin has a role for the regulation of tissue function independently of or synergistically with the systemic (renal) renin signaling [104]. In mice, adrenal renin is normally detected during fetal development, but it disappears at the time of birth [64, 68]. Interestingly, adrenal renin expression can be activated under several conditions. Aldosterone synthase knockout mice show abnormal renin expression in the adrenal gland [71]. In adult rats, renin can be found in glomerulosa cells and appears to be involved in the regulation of aldosterone synthesis. Local renin is upregulated after nephrectomy and after stimulation with Ang-II, high K^+^, and ACTH [30, 61, 105, 141]. The cellular signaling mechanisms translating these conditions and stimuli into increased renin expression, however, are not known. Moreover, it is not clear which pathways are involved to link the loss of Task3 channels to the abnormal renin expression in fasciculata cells and how local renin stimulates steroid hormone secretion.

Why is the hyperaldosteronism phenotype of neonatal *Task3* ^−/−^ mice transient in nature? To address this question, gene chip analyses of 1- and 12-day-old *Task3* ^−/−^ mice were used. The comparison of the results at the two time points revealed several age-dependently expressed genes that are known modulators of adrenal function [7]. For instance, the expression of the nicotinamide nucleotide transhydrogenase, which produces NAPDH as a cofactor for Cyp-enzymes, decreased over time [89, 128]. Similarly, the expression of the store-operated Ca^2+^ channel *Trpc5*, which is possibly involved in the generation of the Ang-II dependent Ca^2+^ signal [57, 74], was decreased in 12-day-old *Task3* ^−/−^ mice. Other factors such as galanin, a neuropeptide stimulating glucocorticoid secretion, showed enhanced expression with age. Most likely, a complex network of factors and pathways counterbalance the cellular deficit induced by the inactivation of *Task3*, and apparently, this compensatory mechanisms take time to fully develop.

## Task1 and Task3 channels serve distinct functions

Task1 and Task3 are related K^+^ channels and probably assemble to form Task1-Task3 heterodimers [25]. One might assume that the two channels serve a similar cellular function. However, the tissue distribution of mRNA expression is not identical and genetic inactivation of each of these channels in mice led to different adrenal phenotypes (Table 2). It appears that an important function of Task1 is to prevent the expression of the aldosterone synthase in fasciculata cells, thereby restricting aldosterone synthase expression to glomerulosa cells. In female *Task1* ^−/−^ mice, the most striking phenotype is the abnormal expression of aldosterone synthase in fasciculata cells that causes severe hyperaldosteronism [55]. In adult *Task1* ^−/−^ males, the presence of Task3 in fasciculata cells may contribute to the correction of this phenotype after puberty, but it is certainly not essential, because adult male *Task1* ^−/−^/*Task3* ^−/−^ mice also display a normal *Cyp11b2* expression pattern [28]. Interestingly, glomerulosa cells of female *Task1* ^−/−^ mice are still sensitive for negative feedback mechanisms in the presence of high plasma aldosterone: they completely shut off aldosterone synthase expression [55].

**Table 2:** Comparison of the different adrenal phenotypes of *Task1* ^−/−^ [55], *Task1* ^−/−^/*Task3* ^−/−^ double knockout [28, 50], *Task3* ^−/−^ [51, 103], and *Task1* ^−/−^/*Dkk3* ^−/−^ double knockout mice [34]

Parameter	Task1^−/−^ ♀+♂ [55]	*Task1* ^−/−^/*Task3* ^−/−^ ♂ [28, 50]}	Task3^−/−^ ♀+♂ [103]	Task3^−/−^ ♂ [51]	Newborn *Task3* ^−/−^ [7]	*Task1* ^−/−^/*Dkk3* ^−/−^ ♀+♂ [34]
Membrane potential of adrenocortical cells under control conditions (compared to wild-type cells; only cells from male mice were analyzed)	Depolarized primary cultured cells	Depolarized glomerulosa cells in adrenal slices	Depolarized primary cultured cells	Glomerulosa cells in adrenal slices were not depolarized	n.a.	Depolarized primary cultured cells; no difference between *Task1* ^−/−^ and *Task1* ^−/−^/*Dkk3* ^−/−^ cells
Cytosolic Ca^2+^	n.a.	n.a.	Spontaneous oscillations in glomerulosa cells (adrenal slices) under control, which were silenced by Ang-II; smaller increase under high K^+^	n.a.	n.a.	n.a.
Aldosterone	Severe hyperaldosteronism in female mice was independent of the salt intake, but normalized by dexamethasone; male mice were normal	Under control conditions hyperaldosteronism in male mice in one study [50]; no hyperaldosteronism in another study [28]; no suppression (even an increase) under high Na^+^ and normal increase under low Na^+^ [28]	Normal aldosterone under control; no suppression under high Na^+^, smaller decrease under low K^+^ in female mice, and normal increase under high K^+^ and low Na^+^; increased aldosterone secretion in perifused adrenal glands (with absent Ang-II)	Mild hyperaldosteronism under control; no suppression under high Na^+^, normal increase under high K^+^ and low Na^+^; higher in vivo Ang-II-dependent aldosterone secretion	Severe hyperaldosteronism; additional increase of corticosterone and progesterone	Hyperaldosteronism in male *Task1* ^−/−^/*Dkk3* ^−/−^ mice, while male Task1^-/-^ had normal aldosterone levels; no further increase by high K^+^
Renin	Decreased plasma renin in female mice under control condition, but not in male mice	Decreased plasma renin in male mice under control [50] or no change [28], low Na^+^, and under high Na^+^ [28]	Decreased plasma renin under control and under high Na^+^; increase under low Na^+^ similar in both genotypes	Decreased plasma renin under control, low Na^+^, and under high Na^+^	Normal plasma renin, but decreased renin in renal lysates; abnormal adrenal renin production	Decreased plasma renin levels in *Task1* ^−/−^/*Dkk3* ^−/−^ mice of both sexes
Aldosterone/renin ratio (ARR)	Increased in female mice	Increased	Increased	Increased	Increased	Increased in male *Task1* ^−/−^/*Dkk3* ^−/−^ mice, while male *Task1* ^−/−^ had normal ARR
Blood pressure (SBP: systolic blood pressure, DBP: diastolic blood pressure)	SBP increased in female mice, but not in male mice; canrenoate normalized SBP	n.a.	SBP was normal under control but increased under high Na^+^	SBP and DBP increased under control; candesartan normalized DBP; SBP increased under high Na^+^	n.a.	n.a.
*Cyp11b2* (aldosterone synthase) expression	Increased *Cyp11b2* mRNA and protein in female mice under control; no further increase of protein under high K^+^	n.a.	*Cyp11b2* mRNA not altered under control	Increased *Cyp11b2* mRNA under control and high Na^+^, protein levels were not increased	Increased *Cyp11b2* mRNA in newborn mice, but not in 12-day-old mice	Increased *Cyp11b2* mRNA in male *Task1* ^−/−^/*Dkk3* ^−/−^ mice, but normal levels in male *Task1* ^−/−^ mice
Aldosterone synthase localization (“zonation”)	Female mice: ectopic expression in zona fasciculata, suppressed expression in zona glomerulosa; normal zonation in male mice	Normal zonation in male mice with glomerulosa-specific expression	Normal zonation in male and female mice with glomerulosa-specific expression	Normal zonation in male mice with glomerulosa-specific expression	Normal zonation with glomerulosa-specific expression	Normal zonation with glomerulosa-specific expression in male *Task1* ^−/−^/*Dkk3* ^−/−^ mice
Plasma electrolytes	Hypokalemia in female mice, normal plasma Na^+^	Hypokalemia	No difference in plasma K^+^ and Na^+^	Hypokalemia	Hypernatremia in 4-day-old mice	n.a.
Additional data	Increased amiloride-sensitive current in distal colon of female mice	–	Sex-dependent *Task3* expression in wt mice (♂: higher than ♀ ZG+ZF; ♀: ZG)	Decreased heart rate at night	Increased expression of Hsd3b6	No effect of Dkk3 knockout in ♀ *Task1* ^−/−^

Please note, that the phenotype of *Task3* ^−/−^ mice was analyzed by two different groups using independent *Task3* ^−/−^ models. The phenotype of *Task1* ^−/−^/*Task3* ^−/−^ mice and of one of the *Task3* ^−/−^ strains [51] was only analyzed in male mice. Aldosterone levels were measured in plasma or in 24 h urine samples. If not stated otherwise, the phenotype of knockout mice is compared with the one of wild-type mice

*n.a.* not analyzed, *ZG* zona glomerulosa, *ZF* zona fasciculata

Probably, the major role of Task3 is setting the resting potential of mouse glomerulosa cells [103]. Also, in rat glomerulosa cells, Task3 homomers seem to be the dominant channel type ensuring the high resting potential [25, 26]. In *Task3* ^−/−^ mice, aldosterone secretion becomes largely autonomous from the renin-angiotensin-axis, because the glomerulosa cells are depolarized even in the absence of Ang-II. Suppression of aldosterone secretion by low K^+^ diet is also compromised, probably due to the inability of *Task3* ^−/−^ cells to hyperpolarize appropriately when extracellular K^+^ is low [103]. In both *Task1* ^−/−^ and *Task3* ^−/−^ mice, the adrenal phenotypes are age-dependent with regard to the ectopic expression of aldosterone synthase and the severe hyperaldosteronism, respectively. In both knockout models, compensation becomes more effective with age and the severity of the symptoms decreases although an abnormal depolarization of the adrenocortical cells is still detectable in cells from adults. In the light of the genetic defects of the K^+^ channel KCNJ5, Ca^2+^ channels, and ion-transporting ATPases that are causative factors for the formation of aldosterone-producing adenomas [42], it is surprising that the depolarized adrenal cortex of *Task1* and *Task3* knockout animals doesn’t show adenomas or overt adrenal hyperplasia. Maybe the adrenal cell biology of *Task1* and *Task3* knockout animals is sufficiently dynamic to allow effective adaptation and prevention of hyperplasia - or the consequences of *Task* channel inactivation are not strong enough to cause obvious hyperplasia during the short lifespan of mice.

## Outlook: role of TASK channels for human adrenal pathology

Na^+^-permeable gain-of-function mutations of *KCNJ5* are causative for some 40 % of aldosterone-producing adenomas [17, 22, 70, 92, 93, 123]. Are *TASK* channels also candidate genes for an increased risk of adrenal hyperplasia or adenoma formation in humans? Interestingly, TASK channels were reported to change their ion selectivity and to become permeable to Na^+^ upon extracellular acidification [86]. However, up to now, no mutations of *TASK* channels have been found that increase the Na^+^ permeability and cause aldosterone-producing adenomas. Perhaps, such permeability-changing *TASK* channel mutations, even if they occur, do not induce proliferation that is a prerequisite for adenoma formation. Loss-of-function mutations of *TASK3* have been linked to Birk Barel mental retardation dysmorphism syndrome [9, 140]. It is, however, not known if these patients have an adrenal phenotype. Interestingly, *TASK3* is a genomically imprinted gene showing paternal silencing [9]. Therefore, a mutation in the maternal copy of TASK3 can lead to a disease, while a mutation in the paternal allele will have no effect. In a genome-wide association study, a correlation of SNPs in the *TASK3* gene with aldosterone levels and the risk for hypertension was found, but no difference between males and females was reported [65]. Also for *TASK1*, a human disease was linked to gene mutations. Loss-of-function mutations can cause pulmonary hypertension [85]. In addition, a single nucleotide polymorphism nearby the TASK1 gene was found to be associated with blood pressure [45], but no adrenal phenotype was reported so far. It is possible that mutations of *TASK* genes can be compensated and do not cause phenotypes strong enough to provoke monogenetic human adrenal diseases. Further studies are required to investigate a potential role of *TASK* channels as modifier genes of adrenocortical disorders.
